# Supplementary material for: Electrically and mechanically driven rotation of polar spirals in a relaxor ferroelectric polymer
Source: Nat Commun. 2024 Jan 8;15:348. doi: 10.1038/s41467-023-44395-5 (PMC10774403; doi:10.1038/s41467-023-44395-5)
Supplement: Supplementary file 1 — Supplementary Information [file 41467_2023_44395_MOESM1_ESM.pdf]

Supplementary Information for

Electrically and Mechanically Driven Rotation of Polar Spirals in a Relaxor Ferroelectric Polymer

Mengfan Guo[1,2,9]*, Erxiang Xu[1,9], Houbing Huang[3,9], Changqing Guo[3], Hetian Chen[1], Shulin Chen[4], Shan He[1], Le Zhou[1], Jing Ma[1], Zhonghui Shen[5], Ben Xu[6], Di Yi[1], Peng Gao[7], Ce-Wen Nan[1], Neil. D. Mathur[2]*, Yang Shen[1,8]*

Affiliations:

[1] State Key Lab of New Ceramics and Fine Processing, School of Materials Science and Engineering, Tsinghua University; Beijing 100084, China.

[2] Department of Materials Science, University of Cambridge; 27 Charles Babbage Road, Cambridge CB3 0FS, UK.

[3] School of Materials Science and Engineering & Advanced Research Institute of Multidisciplinary Science; Beijing Institute of Technology, Beijing 100081, China.

[4] Changsha Semiconductor Technology and Application Innovation Research Institute, College of Semiconductors (College of Integrated Circuits), Hunan University; Changsha 410082, China

[5] International School of Materials Science and Engineering, Wuhan University of Technology; Wuhan 430070, China

[6] Department of Graduate School, China Academy of Engineering Physics; Beijing 100193, China

[7] Electron Microscopy Laboratory and International Center for Quantum Materials, School of Physics, Peking University; Beijing 100871, China.

[8] Center for Flexible Electronics Technology, Tsinghua University; Beijing 100084, China.

[9] These authors contributed equally: Mengfan Guo, Erxiang Xu, Houbing Huang.

[*] E-mail: mg2129@cam.ac.uk (M.G.); ndm12@cam.ac.uk (N.D.M.); shyang_mse@mail.tsinghua.edu.cn (Y.S.)

Table of contents

1. Dielectric properties of relaxor ferroelectric polymer thin films 3

Figures S1 and S2

2. Domain structures of relaxor ferroelectric polymer thin films 5

Figures S3

3. Polarization analysis on polar spirals 6

Figures S4-S7

4. Field-manipulation on polar spirals 11

Figure S8-S12

5. Structure of relaxor ferroelectric polymer thin films 17

Figures S13 and Table S1

6. Theroatical simulation of polar spirals 19

Figures S14-S19; Tables S2 and S3

7. Simulation of the absorption image of polar spirals 23

Figure S20

8. References 24

1. Dielectric properties of relaxor ferroelectric polymer thin films

Temperature-dependent broadband dielectric spectroscope experiments (Fig. S1) were conducted on a precision impedance analyzer (Concept 40, Novocontrol) equipped with temperature controller (Quatro Cryosystem, Novocontrol). The perturbation voltage was set at 0.5 V. Circular copper electrodes (10.4 mm in diameter) were sputtered through a stainless-steel shadow mask and then connected to the analyzer in temperature control chamber. The temperature controller uses two heaters to control the chamber temperature. One is used to generate gas nitrogen with monitored pressure from a Dewar with liquid nitrogen, while another one is used to control the temperature of the nitrogen flow blown to the chamber. During the measurements, the heating rate was usually around 5°C min^{-1}, and the system usually took 15 min to obtain thermal balance before the start of each dielectric test.

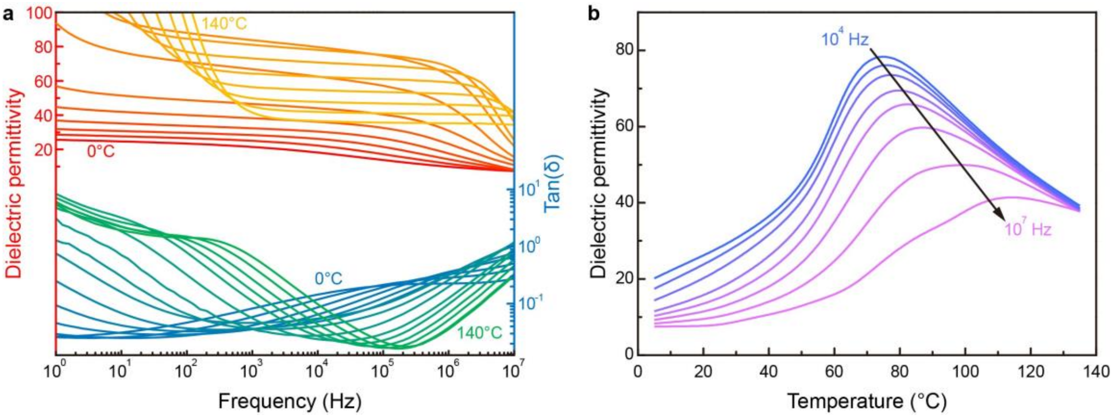

Fig. S1. Temperature-dependent dielectric measurements of the relaxor ferroelectric polymer. a, Temperature-dependent broadband dielectric spectroscope of a thick film of P(VDF-TrFE) with molar ratio of 50/50 from 0°C to 140°C. **b**, Frequency-dependent profiles of dielectric permittivity from 10^4 to 10^7 Hz, showing diffuse dielectric peaks in varied measured frequency.

P-E loops (Fig. S2) were obtained on a Sawyer-Tower circuit (Precision Multiferroic II, Radiant Technologies) by applying a voltage stimulus to the samples. Circular copper electrodes (2.6 mm in diameter) were sputtered through a stainless-steel shadow mask onto films. The P-E loop was collected using dipolar triangular voltage waves with a frequency of 10 Hz.

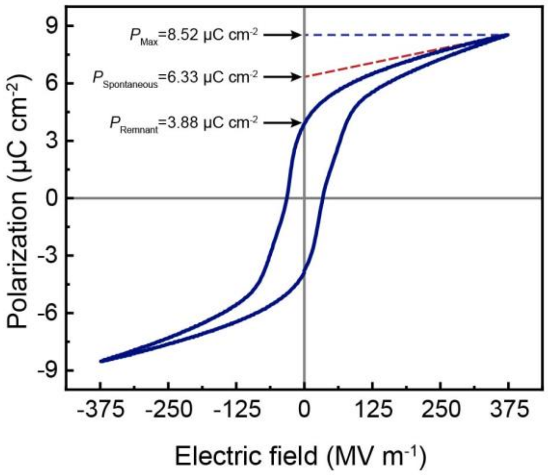

Fig. S2. Electric hysteresis measured in edge-on lamellae thick film of the relaxor ferroelectric polymer. Maximal polarization, spontaneous polarization, and remnant polarization are denoted by the arrows. The spontaneous polarization is further used to calculate the toroidal moment of polar spirals.

2. Domain structures of relaxor ferroelectric polymer thin films

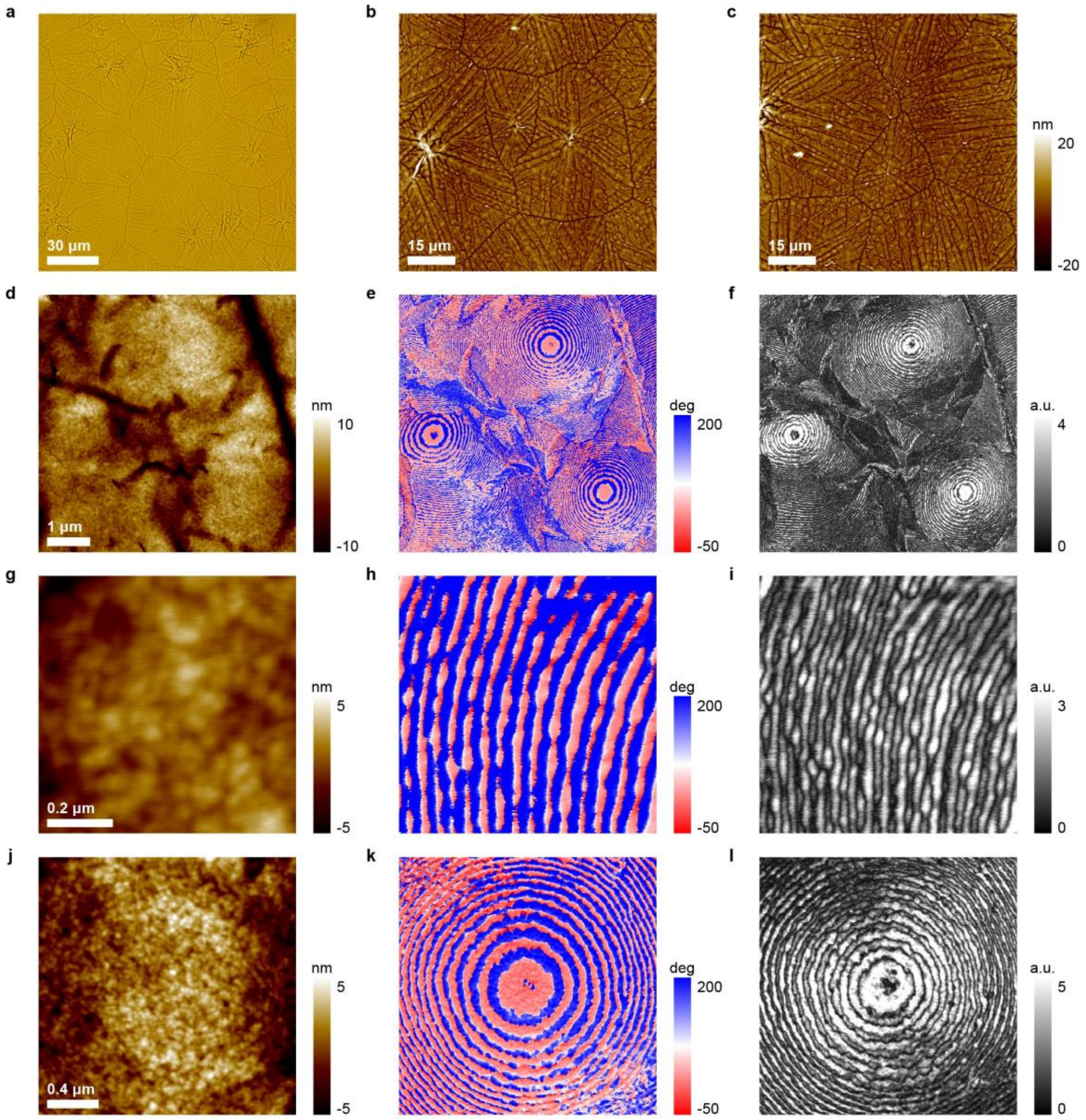

Fig. S3. Morphology and ferroelectric domain structures of face-on lamellae of the relaxor ferroelectric polymer. a, Optic microscopy image of a thin film of the relaxor ferroelectric polymer. The scale bar is 30 µm. **b**, **c**, AFM surface morphology images of two separated regions in thin films of the relaxor ferroelectric polymer. The scale bars are 15 µm. **d**-**f**, AFM morphology (**d**), IP-PFM phase (**e**) and IP-PFM amplitude (**f**) of a region in a relaxor ferroelectric polymer thin film. The domain structure exhibits predominant curly stripe domains and discretized concentric ring-shaped domain microregions. The scale bar is 1 µm. **g**-**i**, AFM morphology (**g**), IP-PFM phase (**h**) and IP-PFM amplitude (**i**) of a region with curly stripe domains. The scale bar is 0.2 µm. **j**-**l**, AFM morphology (**j**), IP-PFM phase (**k**) and IP-PFM amplitude (**l**) of a region with concentric ring-shaped domains. The scale bar is 0.4 µm.

3. Polarization analysis on polar spirals

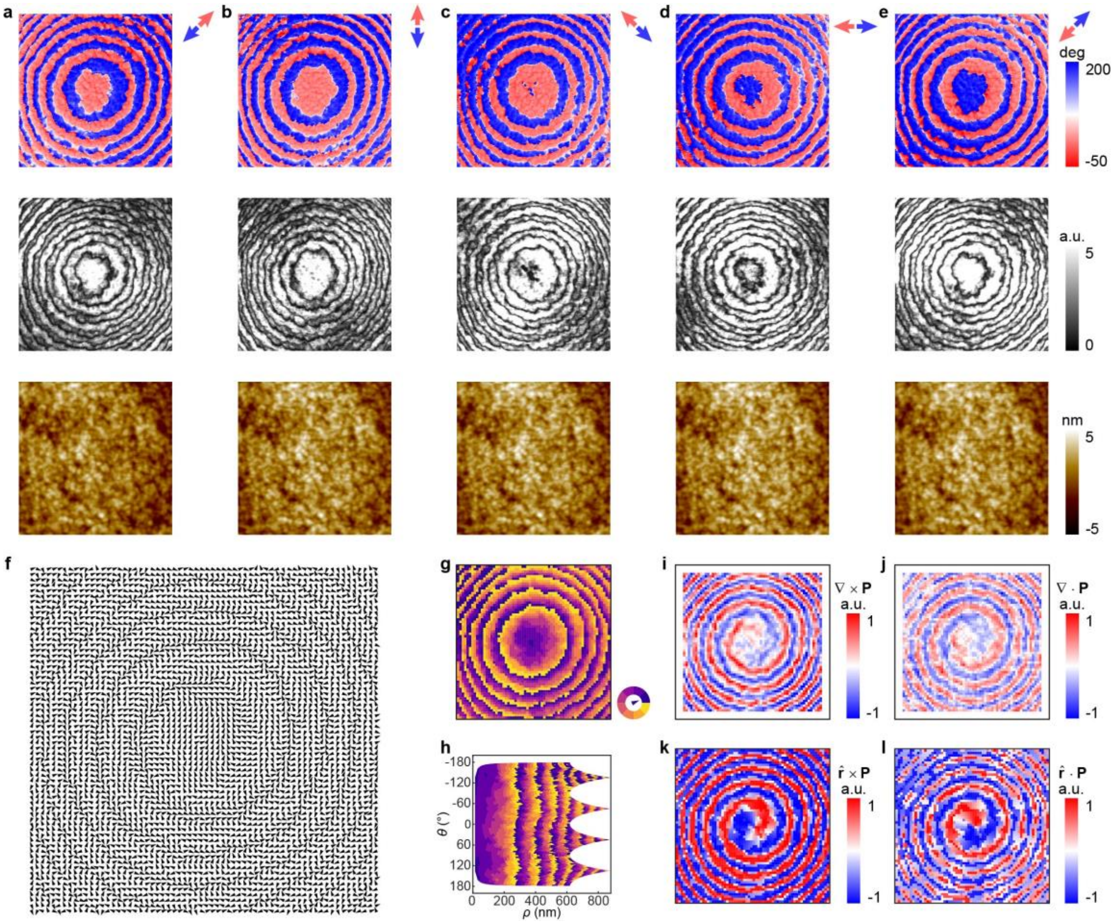

Fig. S4. Angle-resolved IP-PFM measurement and polarization analysis on a CCW polar spiral. a-e, IP-PFM phase, IP-PFM amplitude and AFM morphology images of a CCW polar spiral with the sample rotated by -45° (**a**), 0° (**b**), 45° (**c**), 90° (**d**), and 135° (**e**). Sample rotation along CW direction, which is the measurement axis rotation along CCW direction, is defined with a positive sign. The arrows at the upper right of each figure denote the measurement axes. **f**, **g**, Polarization map of the polar spiral based on arrows (**f**) and colors (**g**), derived from the angle-resolved IP-PFM results (**b-e**). **h**, The distribution of polarization (**f**) in a polar coordinate system. The pole of the polar coordinate is set at the center of **g**. The ρ and θ are the radial and angular coordinates of any point in the system, respectively. **i**, **j**, The curl ($\nabla\times$, **i**) and the divergence ($\nabla\cdot$, **j**) of local polarization (**f**). **k**, **l**, Distribution of normalized electric toroidal moment ($\hat{\mathbf{r}}\times\mathbf{P}$, **k**) and normalized polarization flux ($\hat{\mathbf{r}}\cdot\mathbf{P}$, **l**) of local polarization (**f**). The $\hat{\mathbf{r}}=\mathbf{r}/|\mathbf{r}|$ is a unit displacement vector from the center point to the local point. The center point is adopted from the geometric center point of the Laplace operator (Δ) of local polarization (see Methods for more details). The side length of all the figure is 1.256 μm.

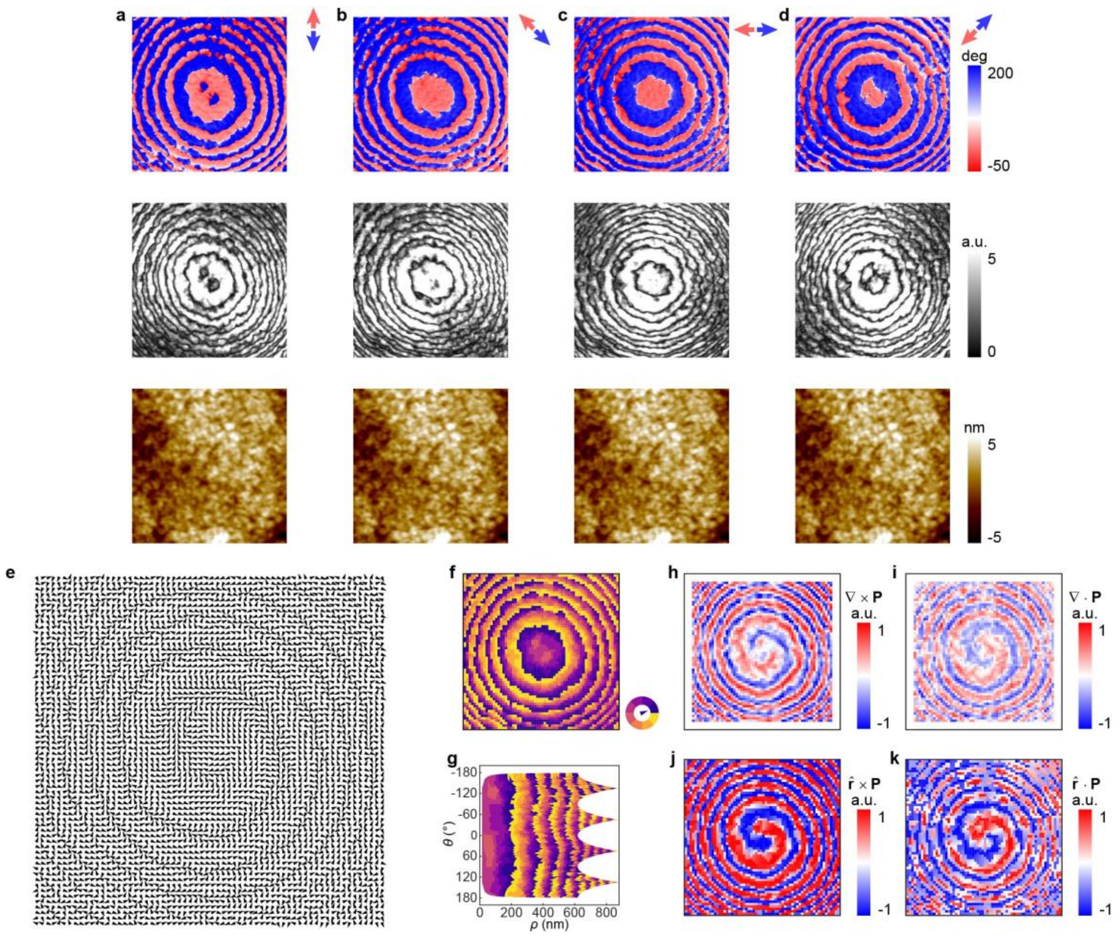

Fig. S5. Angle-resolved IP-PFM measurement and polarization analysis on a CW polar spiral. a-d, IP-PFM phase, IP-PFM amplitude and AFM morphology images of a CW polar spiral with the sample rotated by 0° (**a**), 45° (**b**), 90° (**c**), and 135° (**d**). Sample rotation along CW direction, which is the measurement axis rotation along CCW direction, is defined with a positive sign. The arrows at the upper right of each figure denote the measurement axes. **e**, **f**, Polarization map of the polar spiral based on arrows (**e**) and colors (**f**), derived from the angle-resolved IP-PFM results (**a-d**). **g**, The distribution of polarization (**e**) in a polar coordinate system. The pole of the polar coordinate is set at the center of **f**. The ρ and θ are the radial and angular coordinates of any point in the system, respectively. **h**, **i**, The curl ($\nabla\times$, **h**) and the divergence ($\nabla\cdot$, **i**) of local polarization (**e**). **j**, **k**, Distribution of normalized electric toroidal moment ($\hat{\mathbf{r}}\times\mathbf{P}$, **j**) and normalized polarization flux ($\hat{\mathbf{r}}\cdot\mathbf{P}$, **k**) of local polarization (**e**). The $\hat{\mathbf{r}}=\mathbf{r}/|\mathbf{r}|$ is a unit displacement vector from the center point to the local point. The center point is adopted from the geometric center point of the Laplace operator (Δ) of local polarization (see Methods for more details). The side length of all the figure is 1.256 μm.

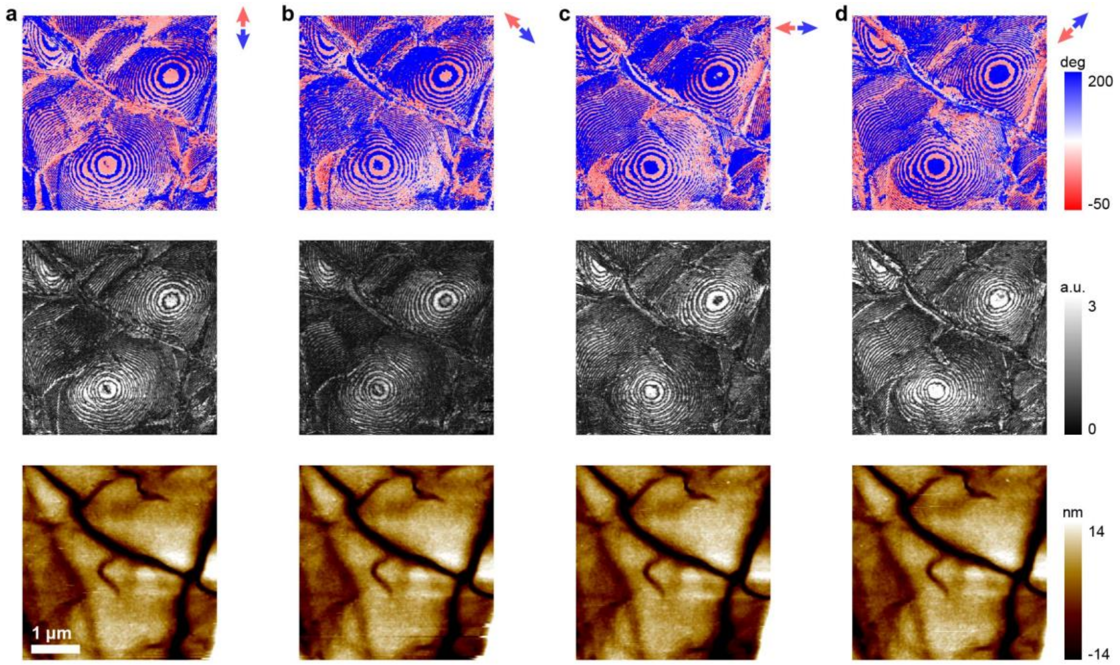

Fig. S6. Observation of CCW and CW polar spirals in the same thin film of relaxor ferroelectric polymer. a-d, IP-PFM phase, IP-PFM amplitude and AFM morphology images of a CW polar spiral (upper right) and a CCW polar spiral (lower left) with the sample rotated by 0° (**a**), 45° (**b**), 90° (**c**), and 135° (**d**). Sample rotation along CW direction, which is the measurement axis rotation along CCW direction, is defined with a positive sign. The arrows at the upper right of each figure denote the measurement axes. The scale bar is 1 μm.

In this study, the electric toroidal moment is calculated to describe the polarization rotation behavior of polar spirals. To comprehensively illustrate this behavior, we calculate both the path-dependent ($\mathbf{G_z}=(\frac{1}{2L})\int_L \mathbf{r}\times\mathbf{P}\,\mathrm{d}L$) and area-dependent ($\mathbf{G_z}=(\frac{1}{2S})\int_S \mathbf{r}\times\mathbf{P}\,\mathrm{d}S$) toroidal moment[S1], as shown in Fig. S7. The integral path and area are a circular ring and circle, respectively, and are centered at the spirals. The path-dependent and area-dependent toroidal moments exhibit similar behaviors. Their values oscillate as integral radius increases, and weigh more on the positive/negative than the other sign for the CW/CCW polar spirals. Since the integral area covers major region of polar spirals, we confirm the emergence of toroidal order in the polar spirals based on its non-zero electric toroidal moment, where CW polar spirals exhibit electric toroidal moments with positive values, and CCW ones exhibit negative values.

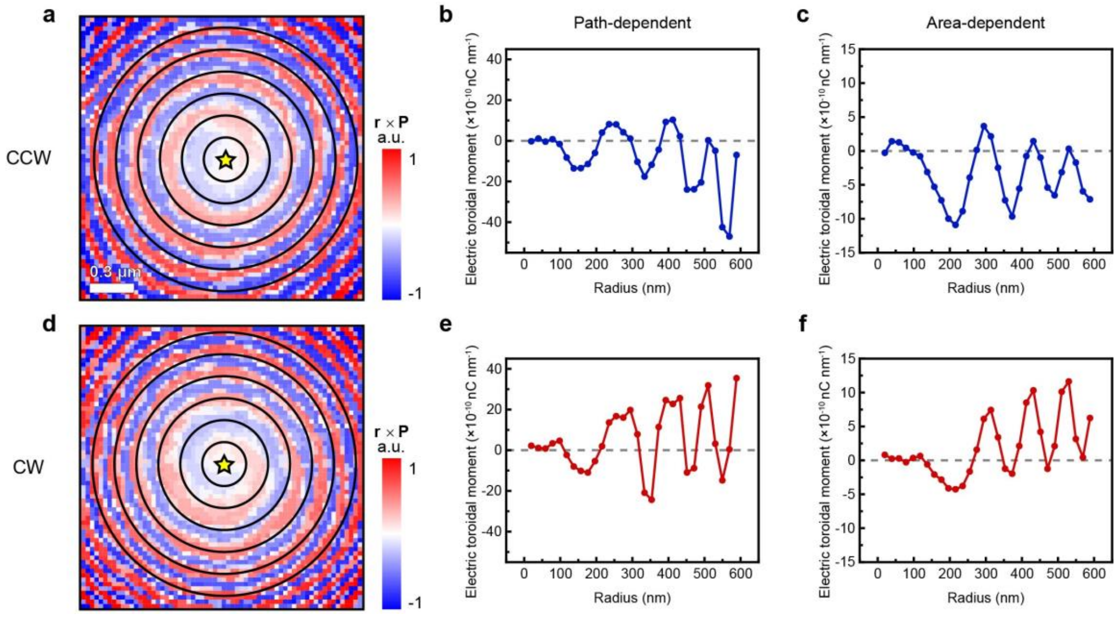

Fig. S7. Electric toroidal moment of CCW and CW polar spirals. a, Distribution of the electric toroidal moment of a CCW polar spiral. The yellow star denotes its geometric center point. The concentric black circles illustrate the circular rings where the electric toroidal moments path integrated, as well as the circular areas where the electric toroidal moments area integrated. The scale bar is 0.3 μm. **b**, **c**, Profile of the path-dependent (**b**) and area-dependent (**c**) electric toroidal moment G_z versus radius of the integrated circle. The grey dashed line denotes the line of G_z=0. **d**, Distribution of the electric toroidal moment of a CW polar spiral. The yellow star denotes its geometric

center point. The concentric black circles illustrate the circular rings where the electric toroidal moments path integrated, as well as the circular areas where the electric toroidal moments area integrated. **e**, **f**, Profile of the path-dependent (**e**) and area-dependent (**f**) electric toroidal moment G_z versus radius of the integrated circle. The grey dashed line denotes the line of G_z=0. The value of local polarization is adopted from the spontaneous polarization deduced from the electric hysteresis of the relaxor ferroelectric polymer in Fig. S2.

4. Field-manipulation on polar spirals

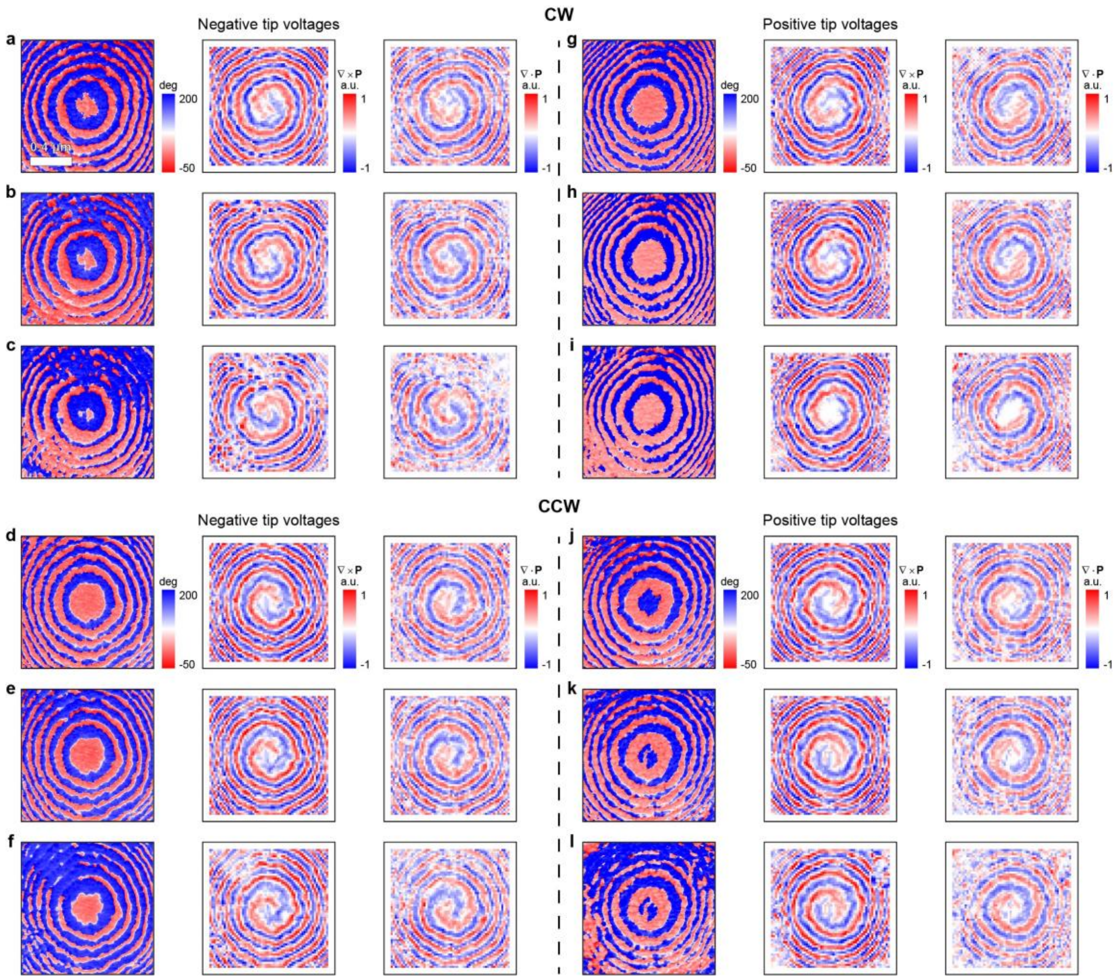

Fig. S8. Electric-field-induced rotation of polar spirals. a-c, IP-PFM phase images, curl of local polarization, divergence of local polarization within a CW polar spiral, before electric-field-induced rotation (**a**), after applying a voltage of -10 V (**b**, electric field of -100 MV m^{-1}, rotation of 37 degree), and after applying a voltage of -12V (**c**, electric field of -120 MV m^{-1}, rotation of 84 degree). **d-f**, IP-PFM phase images, curl of local polarization, divergence of local polarization within a CCW polar spiral, before electric-field-induced rotation (**d**), after applying a voltage of -9 V (**e**, electric field of -90 MV m^{-1}, rotation of -19 degree), and after applying a voltage of -10 V (**f**, electric field of -100 MV m^{-1}, rotation of -60 degree). **g-i**, IP-PFM phase images, curl of local polarization, divergence of local polarization within a CW polar spiral, before electric-field-induced rotation (**g**), after applying a voltage of 13 V (**h**, electric field of 130 MV m^{-1}, rotation of 20 degree), and after applying a voltage of 17 V (**i**, electric field of 170 MV m^{-1}, rotation of 67 degree). **j-l**, IP-PFM phase images, curl of local polarization, divergence of local polarization within a CCW polar spiral, before electric-field-induced rotation (**j**), after applying a voltage of 13 V (**k**, electric field of 130 MV m^{-1}, rotation of -20 degree), and after applying a voltage of 17 V (**l**, electric field of 170 MV m^{-1}, rotation of -47 degree). The scale bar is 0.4 μm.

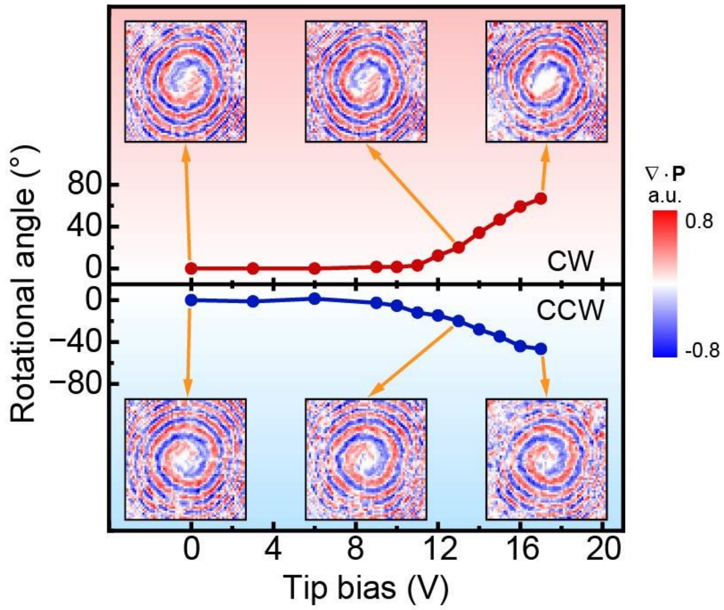

Fig. S9. Electric-field-induced rotation of polar spirals using positive tip voltages. Red dots and line in the upper half denote rotational change of a CW polar spiral, and blue dots and line in the lower half denote rotational change of a CCW polar spiral. The three upper insets are the CW polar spiral rotating along the CW direction, and the three lower insets are the CCW polar spiral rotating along the CCW direction. All insets are the divergence of local polarization. Arrows denote the voltages applied to the polar spirals. The side length of each inset is 1.138 μm.

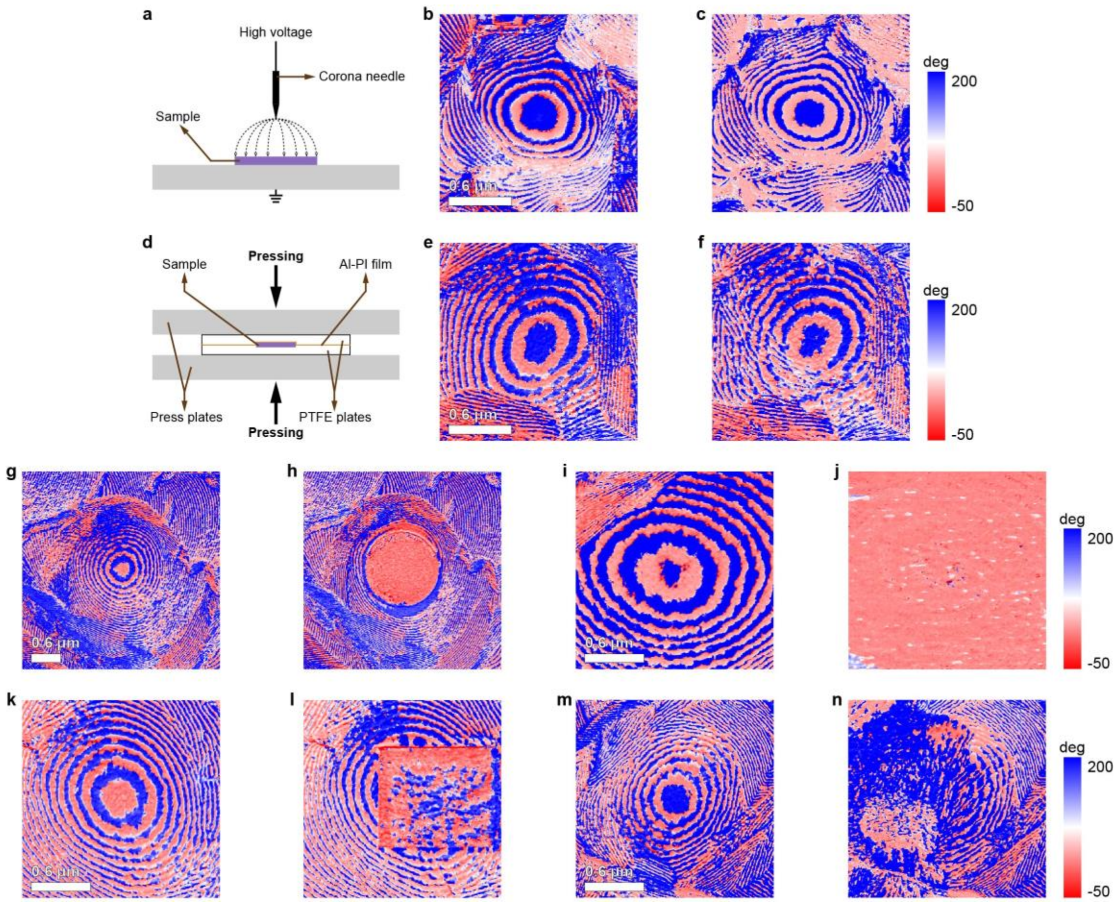

Fig. S10. Macroscopic field-induced rotation and destruction of polar spirals. a, A schematic illustration of how electric field has been macroscopically applied on to the thin film by using corona poling. **b**, **c**, IP-PFM phase images of a polar spiral before (**b**) and after (**c**) the application of macroscopic electric field. **d**, A schematic illustration of how stress has been macroscopically applied on to the thin film by using press machine. **e**, **f**, IP-PFM phase images of a polar spiral before (**e**) and after (**f**) the application of macroscopic stress. **g**, **h**, IP-PFM phase images of a polar spiral before (**g**) and after (**h**) the application of a tip-loaded voltage, yielding a transition to monodomain state. **i**, **j**, IP-PFM phase images of a polar spiral before (**i**) and after (**j**) the application of macroscopically loaded voltage, yielding a transition to monodomain state. **k**, **l**, IP-PFM phase images of a polar spiral before (**k**) and after (**l**) the application of tip-loaded force, yielding a transition to stripe domain state. **m**, **n**, IP-PFM phase images of a polar spiral before (**m**) and after (**n**) the application of a macroscopically loaded force, yielding a transition to stripe domain state. The scale bars are 0.6 μm.

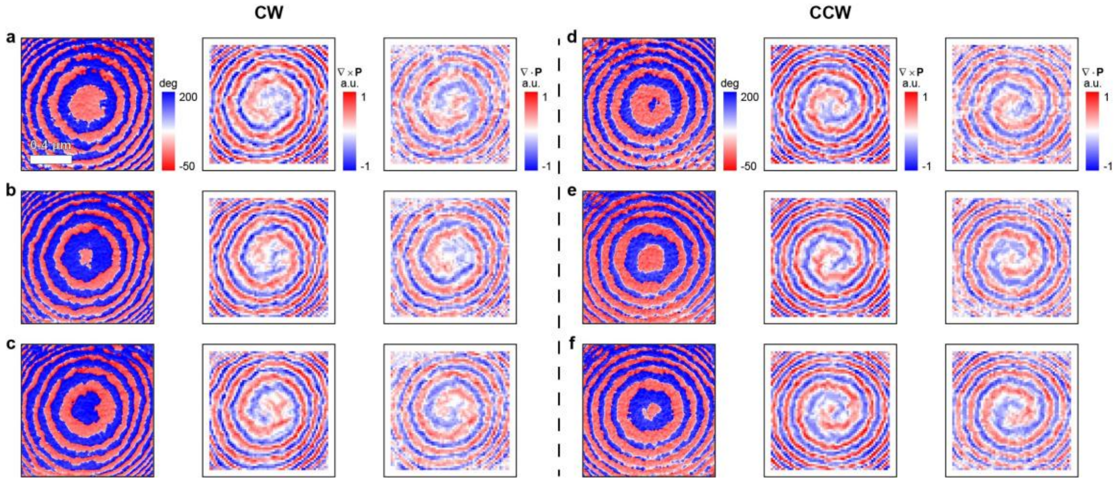

Fig. S11. Stress-induced rotation of polar spirals. a-**c**, IP-PFM phase images, curl of local polarization, divergence of local polarization within a CW polar spiral, before stress-induced rotation (**a**), after applying a force of 18 nN (**b**, stress of 278 MPa, rotation of 90 degree), and after applying a force of 23 nN (**c**, stress of 306 MPa, rotation of 236 degree). **d**-**f**, IP-PFM phase images, curl of local polarization, divergence of local polarization within a CCW polar spiral, before stress-induced rotation (**d**), after applying a force of 19 nN (**e**, stress of 283 MPa, rotation of -84 degree), and after applying a force of 21 nN (**f**, stress of 296 MPa, rotation of -132 degree). The scale bar is 0.4 μm.

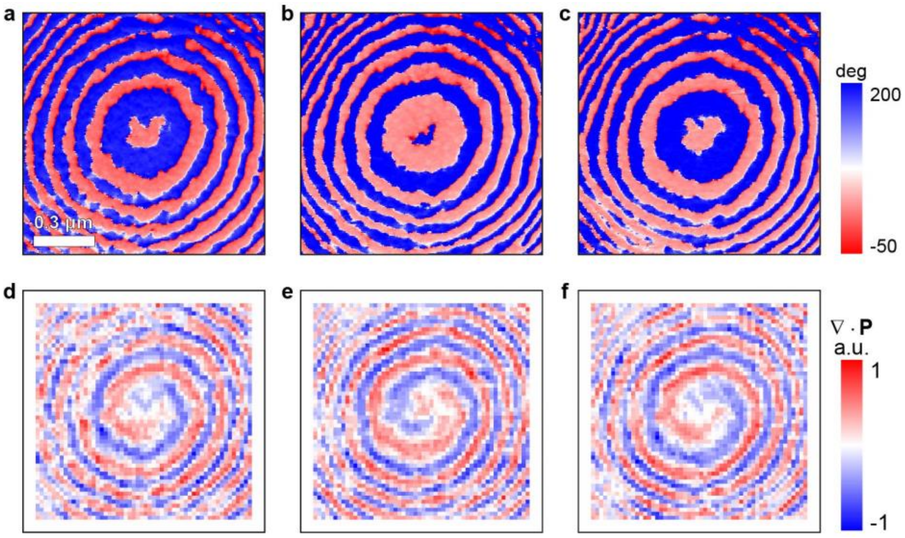

Fig. S12. The reset of polar spirals by rotating 360 degrees. a-c, IP-PFM images of a CW polar spiral before any rotation (**a**), after CW rotation by 225° (**b**) and further CW rotation by 127° (352° in total) (**c**). The scale bar is 0.3 µm. **d-f**, The divergence of local polarization in the same region as **a-c** before any rotation (**d**), after CW rotation by 225° (**e**) and further CW rotation by 127° (352° in total) (**f**).

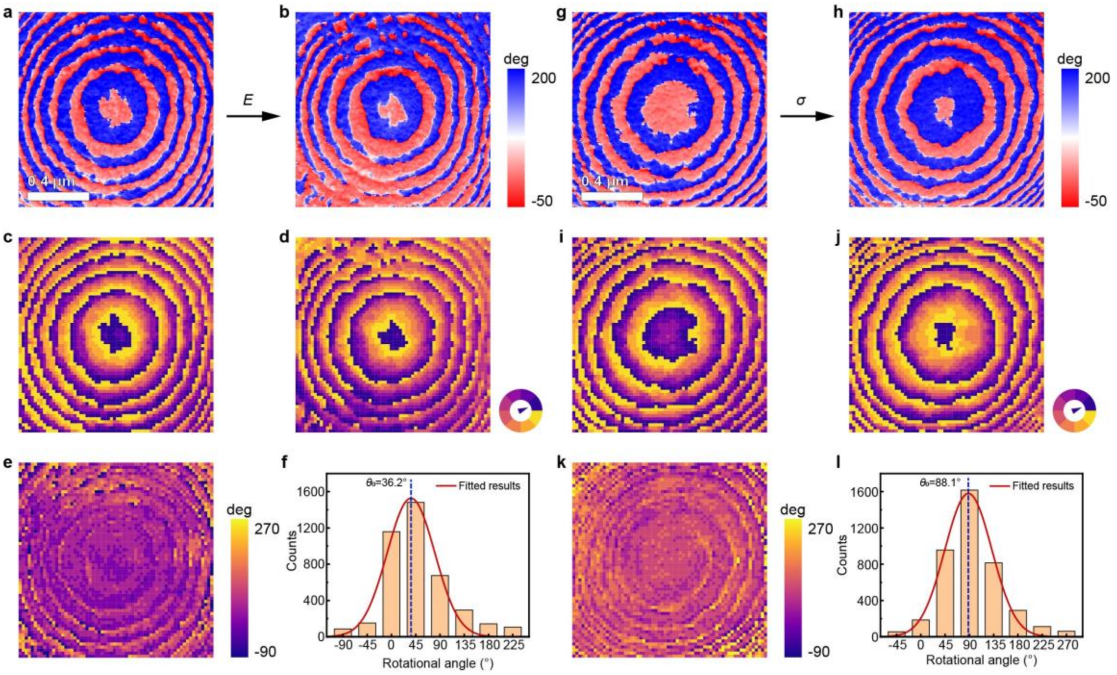

Fig. S13. Field-induced collective rotation of local polarizations within polar spirals. a, **b**, IP-PFM phase images of a polar spiral before (**a**) and after (**b**) electric-field-induced change. **c**, **d**, Polarization color maps of the same region as **a**-**b** before (**c**) and after (**d**) electric-field-induced change. **e**, The distribution of rotational angle of each local polarization during the electric-field-induced change (**c** to **d**). **f**, The histogram of rotational angle and a fitting to the normal distribution yielding a Gaussian peak centered at 36.2°. **g**, **h**, IP-PFM phase images of a polar spiral before (**g**) and after (**h**) stress-induced change. **i**, **j**, Polarization color maps of the same region as **g**-**h** before (**i**) and after (**j**) stress-induced change. **k**, The distribution of rotational angle of each local polarization during the stress-induced change (**i** to **j**). **l**, The histogram of rotational angle and a fitting to the normal distribution yielding a Gaussian peak centered at 88.1°. The scale bars are 0.4 μm.

The visualization in Supplementary Movies 4 and 5 is produced under the assumption that local polarizations collectively rotate during the field manipulation. The fitted rotational angles of the polar spiral were first extracted from Supplementary Movies 2 and 3, and were then added to the polarization maps. The divergence of local polarization in each state was calculated and put together in the movie. Extra frames with the ending picture have been added at the end to emphasize the non-volatile feature.

5. Structure of relaxor ferroelectric polymer thin films

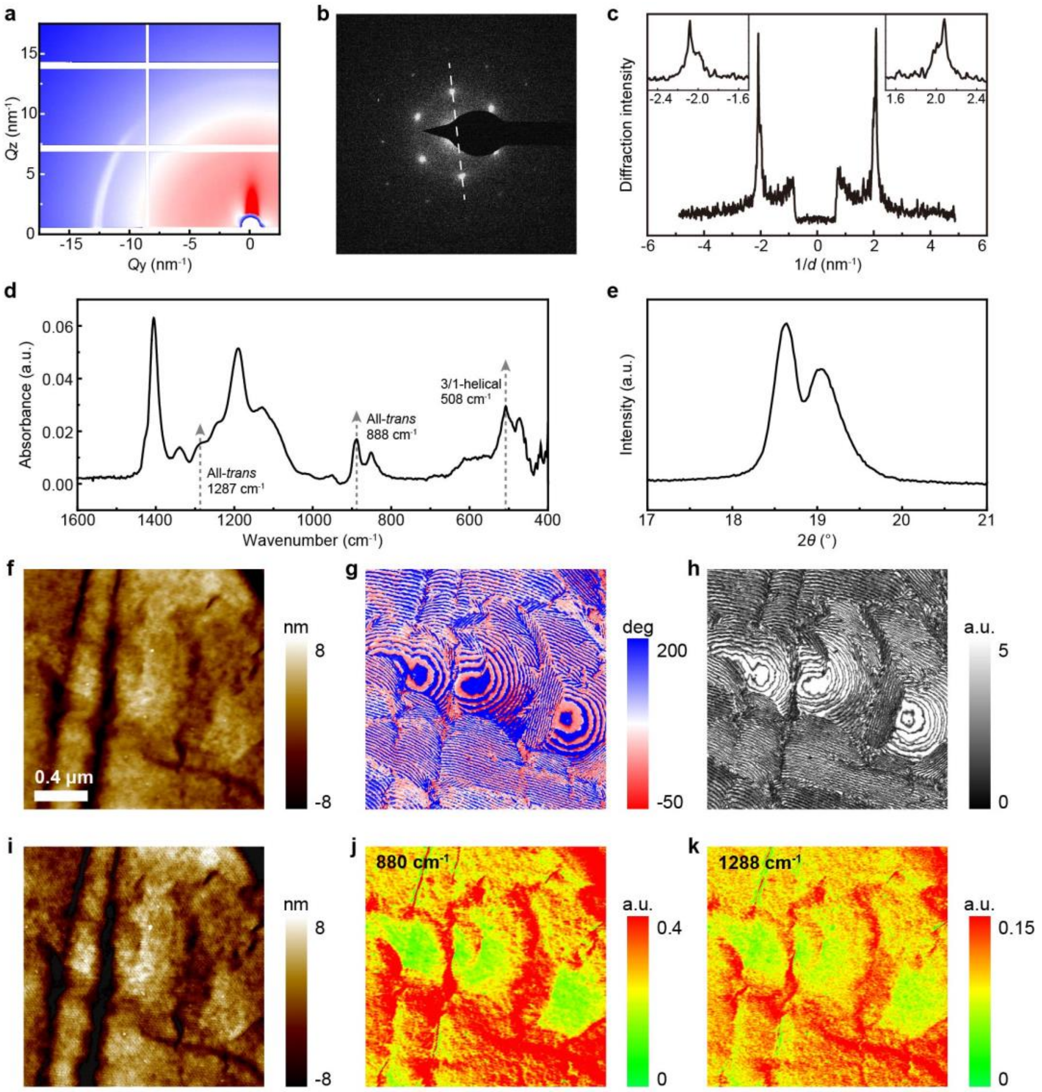

Fig. S14. Structural characterizations on the relaxor ferroelectric polymer thin films. **a**, GI-WAXS pattern of the face-on lamellae thin film, with main scattering arc at Q_y axis. **b**, SAED pattern in a face-on lamellae thin film. The aperture diameter is 3.8 μm. **c**, The profile of diffraction intensity along the white dashed line in **b**. The two insets show the detailed peak intensity splitting of the two diffraction spots. **d**, FTIR profile of a face-on lamellae thin film. Grey dashed lines denote the characteristic peaks at 1287 and 888 cm^{-1} corresponding to the *trans*-planar phase, and at 508 cm^{-1} corresponding to the 3/1-helical phase. **e**, XRD diffraction of an edge-on lamellae thin film. The split peaks at 2θ=18.64° and 2θ=19.04° correspond to the diffraction of (110)/(200) lattice plane in the 3/1-helical phase and the *trans*-planar phase, respectively. **f-h**, AFM morphology (**f**), IP-PFM phase (**g**) and IP-PFM amplitude (**h**) of three isolated polar spirals. **i**, AFM morphology image from AFM-IR measurements, showing the same region as **f**. **j**, **k**, AFM-IR absorption images in **i**, with an IR beam

polarized along OOP direction at 880 cm^{-1} (**j**) and 1288 cm^{-1} (**k**). The scale bar is 0.4 μm.

Table S1. Reference and measured (110)/(200) spacing of *trans*-planar and 3/1-helical phases in face-on lamellae thin films of the relaxor ferroelectric polymer.

	trans-planar phase	3/1-helical phase
2θ from XRD (°)	19.04	18.64
Q_y from GI-WAXS (nm^{-1})	13.49	13.15
Reference (110)/(200) spacing (nm)	0.4661	0.4760
Measured (110)/(200) spacing (nm)	0.4658	0.4778
Strain	-0.06%	0.37%

6. Theroatical simulation of polar spirals

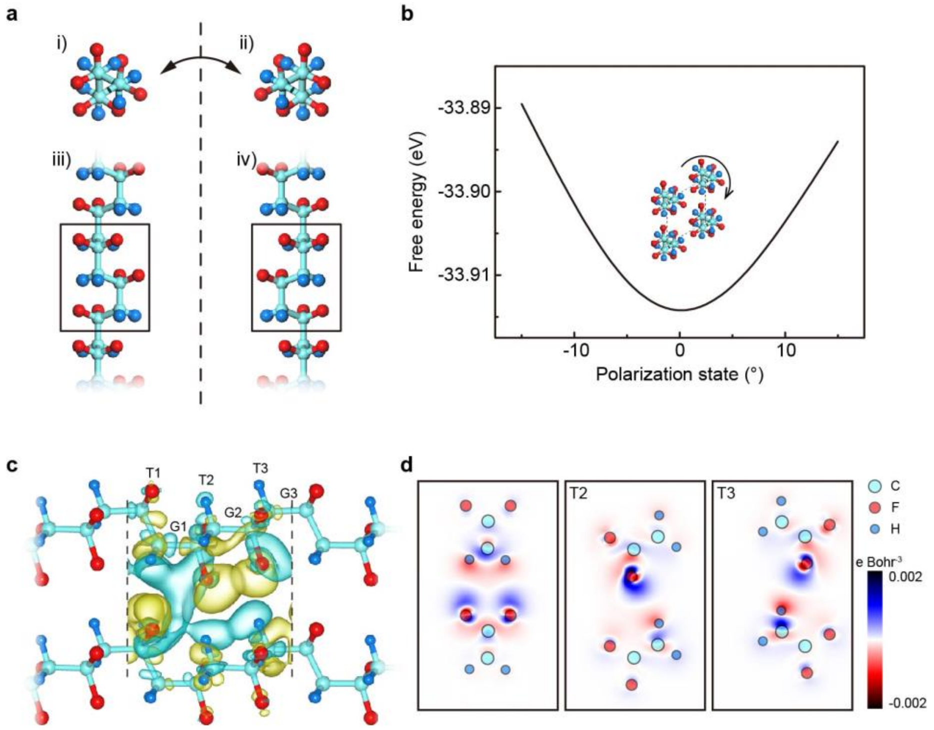

Fig. S15. First-principle calculations on 3/1-helical phase of relaxor ferroelectric polymer. a, Helicity in the 3/1-helical phase. i, ii, Top view of the 3/1-helical chains with $(TG)_3$ (i) and $(T\bar{G})_3$ (ii) conformational sequence. iii, iv, Side view of the 3/1-helical chains with $(TG)_3$ (iii) and $(T\bar{G})_3$ (iv) conformational sequence. Boxes denote the periodicity in three -CH_2-CF_2- segments of the helical chains. The dashed line and double-sided arrow denote that the $(TG)_3$ and $(T\bar{G})_3$ chains are mirrored to each other. For convenience, PVDF chains instead of P(VDF-TrFE) chains are used for illustration. **b**, Free energy of lattice of $(TG)_3$ helical chains versus polarization state. The free energies are calculated based on $(TG)_3$ helical chains in their lattice with collective rotation. The inset shows the lattice of the $(TG)_3$ chains. **c**, **d**, Charge density difference analysis on the helical chain pair. **c**, Three-dimensional charge density difference analysis on the pair of $(TG)_3$ helical chains with the polarization state exhibiting the global lowest energy. Isosurface denoting charge accumulation (yellow) and charge depletion (blue) is drawn around the chains. The conformations of each C-C bonding in the backbone are denoted. **d**, Two-dimensional sections of charge density difference at each trans C-C bonding denoted in **c**. Section at T1 exhibits an intermolecular interaction with its spatial alignment perpendicular to both chains, similar to those observed in trans-planar phase. Instead, sections at T2 and T3 exhibit tilted intermolecular interaction. The red, blue, and cyan atoms are F, H, and C, respectively.

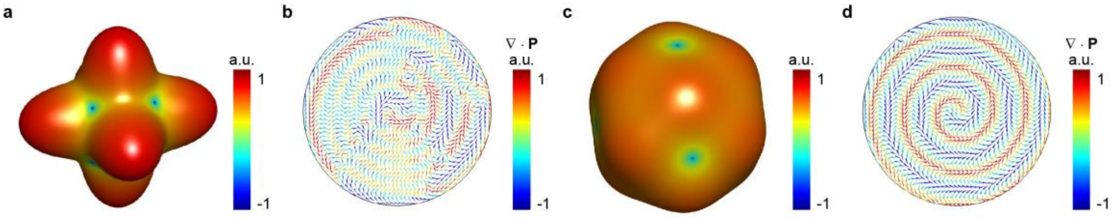

Fig. S16. Phase-field simulations with strong and weak anisotropy. a, **b**, The Landau energy surface with strong anisotropy (**a**) and simulated polarization map (**b**). **c**, **d**, The Landau energy surface with weak anisotropy (**c**) and simulated polarization map (**d**). The dielectric stiffness coefficients that determine the anisotropy of Landau energy surface are included in Supplementary Tables 2 and 3. The color of vectors depicts the divergence of local polarization.

Table S2. Values of parameters used for phase-field simulations on systems with strong anisotropy (at 25°C).

Parameter	Value	Parameter	Value
α_1 (J m C^{-2})	$1.412(T\text{-}42)\times10^5$	Q_{11} (m^4 C^{-2})	-0.0162
α_{11} (J m^5 C^{-4})	-1.842×10^9	Q_{12} (m^4 C^{-2})	0.0441
α_{111} (J m^9 C^{-6})	2.585×10^{11}	Q_{44} (m^4 C^{-2})	-0.12
c_{11} (J m^{-3})	4.88×10^{10}	G_{11} (J m^3 C^{-2})	9.96×10^{-10}
c_{12} (J m^{-3})	5.6×10^9	G_{12} (J m^3 C^{-2})	0
c_{44} (J m^{-3})	2.16×10^{10}	G_{44}/G'_{44} (J m^3 C^{-2})	4.98×10^{-10}

Table S3. Values of parameters used for phase-field simulations on systems with weak anisotropy (at 25°C).

Parameter	Value	Parameter	Value
α_1 (J m C^{-2})	$1.412(T\text{-}42)\times10^5$	c_{44} (J m^{-3})	2.16×10^{10}
α_{11} (J m^5 C^{-4})	-1.842×10^8	Q_{11} (m^4 C^{-2})	-0.0162
α_{12} (J m^5 C^{-4})	-1.4736×10^9	Q_{12} (m^4 C^{-2})	0.0441
α_{111} (J m^9 C^{-6})	2.585×10^{12}	Q_{44} (m^4 C^{-2})	-0.12
α_{112} (J m^9 C^{-6})	9.6×10^{12}	G_{11} (J m^3 C^{-2})	9.96×10^{-10}
α_{123} (J m^9 C^{-6})	1.0857×10^{13}	G_{12} (J m^3 C^{-2})	0
c_{11} (J m^{-3})	4.88×10^{10}	G_{44}/G'_{44} (J m^3 C^{-2})	4.98×10^{-10}
c_{12} (J m^{-3})	5.6×10^9		

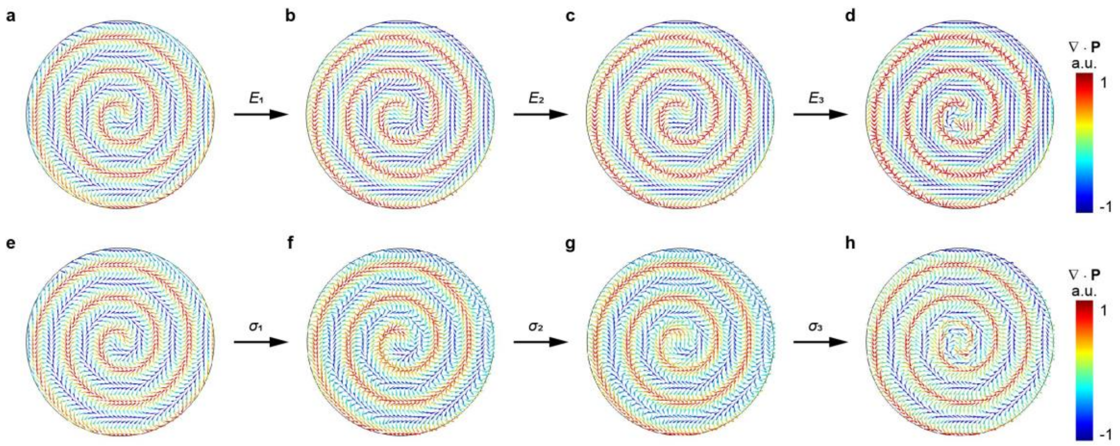

Fig. S17. Phase-field simulations of CCW polar spirals in a thin film. a-d, Polarization maps within a CCW polar spiral in its initial state (**a**) and after the application of downward electric field of 1.00 kV m^{-1} (**b**), 3.23 kV m^{-1} (**c**), and 15.00 kV m^{-1} (**d**). **e-h**, Polarization maps within a CCW polar spiral in its initial state (**e**) and after the application of compressive stress of 2.64 MPa (**f**), 3.60 MPa (**g**), and 12.0 MPa (**h**). The color of vectors depicts the divergence of local polarization.

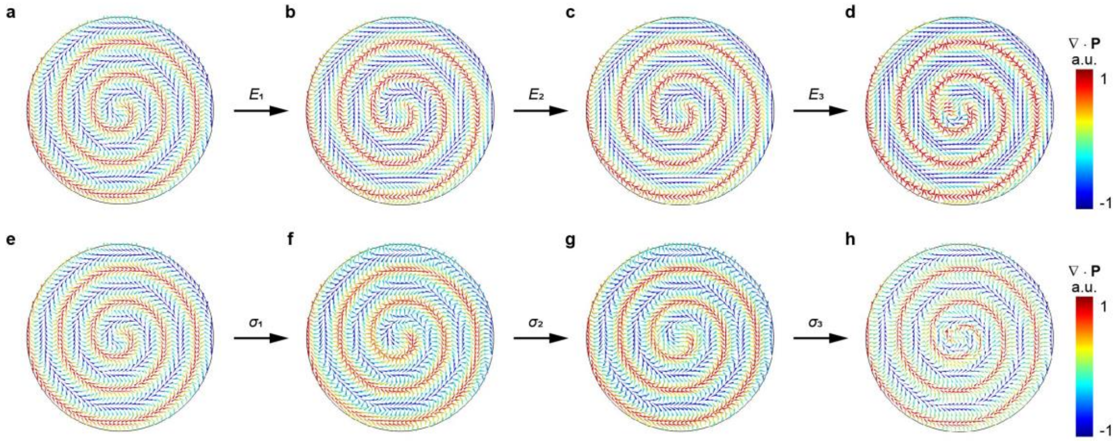

Fig. S18. Phase-field simulations of CW polar spirals in a thin film. a-d, Polarization maps within a CW polar spiral in its initial state (**a**) and after the application of downward electric field of 1.00 kV m^{-1} (**b**), 3.97 kV m^{-1} (**c**), and 15.00 kV m^{-1} (**d**). **e-h**, Polarization maps within a CW polar spiral in its initial state (**e**) and after the application of compressive stress of 2.64 MPa (**f**), 3.60 MPa (**g**), and 12.0 MPa (**h**). The color of vectors depicts the divergence of local polarization.

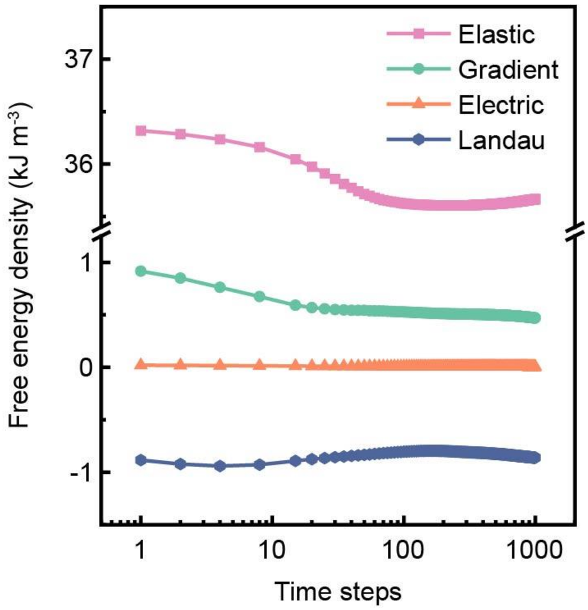

Fig. S19. Time-dependent energy evolution from phase-field simulations on stress-induced changes within a polar spiral. The elastic energy is denoted by pink squares and line. The gradient energy is denoted by green circles and line. The electric energy is denoted by orange triangles and line. The Landau energy is denoted by blue hexagons and line.

7. Simulation of the absorption image of polar spirals

To obtain a predicted distribution of IR absorption in polar spirals, we assumed an IR beam polarized along the IP directions would be projected onto the sample with an intersectional angle of 80° with the vertical direction. Then we calculated the absolute value of sine of the intersection angle between each local polarization and the beam direction. The simulated results in Fig. S20 exhibit concentric and periodic absorption pattern similar to the IR absorption images obtained from AFM-IR measurements.

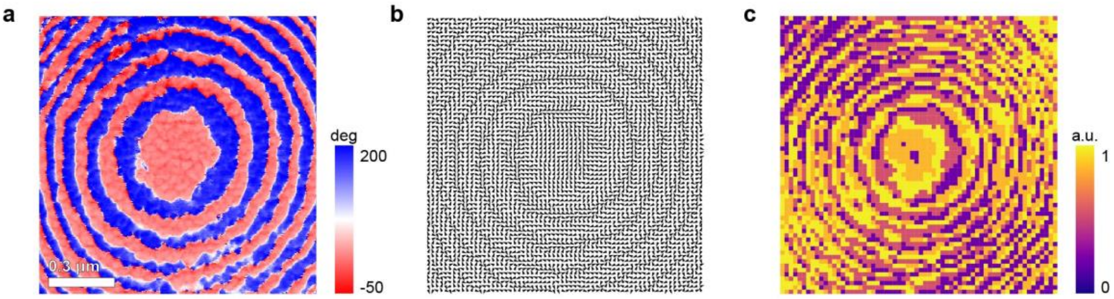

Fig. S20. Predictions on local IR absorption in polar spirals. a, **b**, IP-PFM phase image (**a**) and polarization map (**b**) of a polar spiral. The scale bar is 0.3 μm. **c**, Predicted distribution of absorption on a IR beam polarized along the IP directions within the polar spiral.

8. References

S1. Naumov, I. I., Bellaiche, L. & Fu, H. Unusual phase transitions in ferroelectric nanodisks and nanorods. *Nature* **432**, 737-740 (2004).
