# Supplementary material for: Electrically and mechanically driven rotation of polar spirals in a relaxor ferroelectric polymer
Source: Nat Commun. 2024 Jan 8;15:348. doi: 10.1038/s41467-023-44395-5 (PMC10774403; doi:10.1038/s41467-023-44395-5)
Supplement: Supplementary file 3 — Description of Additional Supplementary Files [file 41467_2023_44395_MOESM3_ESM.pdf]

Description of Additional Supplementary Files

Supplementary Movie 1

Domain patterns of polar spirals with rotated measurement axes and schematics for the polar spirals. a, IP-PFM domain patterns and schematic of a CCW polar spiral varying with rotated measurement axes, exhibiting collective outward domain-wall shifting. b, IP-PFM domain patterns and schematic of a CW polar spiral varying with rotated measurement axes, exhibiting collective inward domain-wall shifting. Sample rotated along CW direction in the angle-resolved PFM measurements, which meant the measurement axis rotating along CCW direction.

Supplementary Movie 2

The evolution of domain pattern of an electric-field-manipulated polar spiral. The evolution of IP-PFM phase (left) and IP-PFM amplitude (right) images of a polar spiral with increasing electric field, exhibiting collective inward domain-wall shifting.

Supplementary Movie 3

The evolution of domain pattern of a stress-manipulated polar spiral. The evolution of IP-PFM phase (left) and IP-PFM amplitude (right) images of a polar spiral with increasing stress, exhibiting collective inward domain-wall shifting.

Supplementary Movie 4

Visualization of the non-volatile and multistate rotation of the polar spiral with increasing electric field. The evolution of the divergence of local polarization in the polar spiral with increasing electric field under the assumption that local polarizations collectively rotate. Extra frames with the ending picture have been added at the end to emphasize the non-volatile feature.

Supplementary Movie 5

Visualization of the non-volatile and multistate rotation of the polar spiral with increasing stress field. The evolution of the divergence of local polarization in the polar spiral with increasing stress field under the assumption that local polarizations collectively rotate. Extra frames with the ending picture have been added at the end to emphasize the non-volatile feature.
